# Progress in Psychiatry

**Published:** 1901-06-22

**Authors:** 


### PROGRESS IN PSYCHIATRY.

(*Continued from page* 102.)

**Encephalopathy and Insanity from Recent Syphilis.**—The cerebral implications of syphilis usually belong to the late secondary or the tertiary periods. A few earlier cases have been noted by Lancereaux, Heubner (one case with depressive mental symptoms one month after infection), and others. Fournier and Hutchinson have both noted cases with grave cerebral disturbances between the first and second year after syphilitic infection. Beaudoin collected from Fournier's Clinic in Paris twenty-six cases of cerebral syphilis in which the symptoms declared themselves in from three to eighteen months after the appearance of the primary sore. Rumpf[84] records forty cases of cerebral syphilis, in nine of which the symptoms appeared during the first year after infection. Neumann also gives several cases with recent cranial nerve palsies, ophthalmoplegia, aphasia, epilepsy, and optic neuritis occurring in the first year of syphilis. At a discussion at the Royal Medico-Chirurgical Society of London in March, 1891, similar cases of syphilitic encephalopathy were mentioned by Lydston.[85] Althaus[86] has given two cases, one of cerebral syphilis with hemiplegia occurring eight months after infection, and another of syphilitic headache, epileptic fits, and hemiplegia three months after infection. Pick[87] records the case of a man, aged 58 years, who six months after syphilitic infection, and while showing the secondary rash, had also headache, disturbance of sensibility of the right half of the face (trigeminal nerve), disturbance of speech and dulness of hearing. Then followed paralysis of the facial and hypoglossal nerves of the right side, visual disturbances, and right-sided hemiplegia. Later on he gradually developed other symytoms and died of coma. The necropsy showed a recent softening in the posterior part of the right internal capsule, *endarteritis obliterans* in the region of the right Sylvian fosse, and gummatous meningitis of the ventral surface of the pons. There were also miliary neoplasms (granulomata) in the inner surface of the dura mater. Lannois and Fournier[88] also record a similar case. In a recent article Mingazzini[89] gives five cases of his own illustrating the occurrence of syphilitic encephalopathy after recent infection, one of which is of special interest and is as follows: A man, aged 50 years, a naval officer, was free from hereditary neurotic taint and from alcoholism. He contracted syphilis in July 1896, and the sore healed in a month, but after that he became very depressed in spirits, and even attempted suicide with a revolver. He had two separate attacks of delirium with severe headache, and nocturnal emissions of semen. The following year there were attacks of depression mixed with exaltation. The headache was associated with pains in the bones of the body, and there was swelling of one testicle. A little bronchitis set in as a complication. All this time he was treated with iodide of potassium. The headache continued for thirteen months, and then followed ptosis of the right eyelid. Other partial paralyses (facial and hypoglossal) now followed. The headache was now mainly occipital in position. Romberg's symptom now appeared, and finally he became apathetic, developed optic neuritis, hemiplegia, inequality of the pupils, and glosso-kinæsthetic aphasia, dying fourteen months after the infection. The autopsy revealed a gumma in the left testicle, *endarteritis obliterans* in the right and left vertebrals, the basilar, the left Sylvian artery, and at the posterior perforated space. There was partial thrombosis of these arteries. A large red softening was found affecting the head and body of the candate nucleus and involving the lenticular nucleus, external capsule, and the pyramidal tract within the capsule. There were also present adhesions of the dura mater to the right oculo-motor nerves.

MacBride,[90] in referring to the symptoms of cerebral syphilis, draws attention to the fact that in the majority of cases the mental symptoms are largely of a negative kind—*e.g.*, loss of mental power and of self-control, impairment of memory, loss of interest in surroundings, and a liability to undue emotional disturbances such as anger or crying. Attacks of loss of speech or of sight ("blind spells") occur not uncommonly at an early stage. Delusions, if present in an early stage of the disease, are incongruous and of fleeting and changeable character, and never deeply influencing character or conduct. There is general confusion of mental processes. Drinking and drug habits are likely to be resorted to at this stage, the patient seeking an anodyne for his mental distress. It is found that syphilitic inebriates are particularly liable to commit suicide after a drinking bout. Syphilitic insanity sometimes closely resembles general paralysis, but has more frequent focal symptoms—*e.g.*, paralysis of one or more cranial nerves, of local cerebral hæmorrhages, neoplasus, or softenings. Charcot long ago called attention to a characteristic of cerebral syphilis—viz., irregular and temporary attacks of aphasia, a condition which is related to the atheroma and disease of the cerebral arteries present in syphilis. In the case of a patient under the care of Dr. MacBride with cerebral syphilis, who would take a long walk or play tennis there followed transient spasm in the right arm and leg followed by temporary paralysis. If the patient talked for any length of time he developed a temporary ataxic aphasia, "showing what little reserve power was left to the brain as the blood-stream was gradually shut down" by the development of endo-ateritis and atheroma. While neurotic predisposition had much to do with determining a special encephalopathy following syphilis, yet it was probable that a special virulence of the syphilitic virus was sometimes the main determining cause; as in the case mentioned by a French writer of a woman who infected five youths with syphilis, with the result that three of them developed and died from general paralysis, while two suffered from cerebral syphilis. Syphilis of the brain occurring after 10 years from the date of infection was rare. Fournier mentions the case of a patient who was entirely well for 17 years after syphilitic infection and then died from cerebral syphilis.

In some instances the cerebral disturbances which may develop within a few years of syphilitic infection closely simulate those of dementia præcox or of general paralysis of the insane, and differential diagnosis is almost impossible. In addition to vesanic manifestations—*i.e.* mental disturbances of the nature of melancholia, mania, acute mental confusion—there is present a degree of mental torpor and stupidity which is unmistakable, and which has led Fournier and others to group such cases under the title of syphilitic pseudo-dementia. Such cases clinically are related to dementia præcox. The expression "syphilitic pseudo-general paralysis" was proposed in 1879 by Fournier, but the morbid entity had already been recognised earlier and described by Zambaco (1862), Lancereaux (1873), and Mickle (1877). Clinical observation shows that certain points of difference exist between this form of disease and the classical form of general paralysis; and, indeed, Fournier at one time formulated these differences as follows: The delusions of syphilitic pseudo-paresis are nearly always free from the grandiose and ambitious character which marks those of true general paralysis; tremor of the tongue and upper lip is less common in pseudo-paresis; the cerebral and cranial nerve palsies are more common and more marked; the apoplectic strokes and sudden paralysis present themselves at an early stage; while the mental degeneration appears later, and cure, though not usual or frequent, is yet possible. More recent researches, however, have served to show that varying grades and degrees of pseudo-paresis may occur, marking every step between early syphilitic encephalopathy and late para-syphilitic encephalopathy or true general paralysis, and in his more recent publications[91] Fournier says that he regards the series of syphilitic and para-syphilitic cerebral affections as forming a continuous whole. Pseudo-paresis may, like true general paresis, present itself under any psychological form, demented, depressive, or expansive. It may, however, unlike true general paralysis, react well to anti-syphilitic treatment (mercurials and iodides), and from this fact, viz., its relative curability, we may regard the pathological conditions present as those of a neoplastic character (granulomata and gummata of the cerebral meninges and vessels), and of rapid evolution, acting by causing mechanical pressure and local vascular congestions which, however, are readily relieved by the treatment adopted.

[84] Die Syphil. Erkrank. des Nervensyst. Wiesbaden, 1887. [85] Journ. of the Amer. Med. Assoc., Fed. 9, 1895, and succeeding numbers. [86] Brit. Med. Jour., 1891, vol. i., p. 485. [87] Zeitsch. für Heilkunde, Bd. xiii., Heft iv. and v. [88] Revue de Medec., 1896, p. 51. [89] Monatschr. für Psychiatrie und Neurolog, Feb. 1899. [90] Journ. of the Amer. Med. Assoc., Feb. 2, 1901. [91] Les infections parayphilitiques, N.D., Paris 1899.

### PROGRESS IN DISEASE OF DIGESTIVE ORGANS.

(*Continued from page* 183.)

